# Primary Immunodeficiency Diseases in Highly Consanguineous Populations from Middle East and North Africa: Epidemiology, Diagnosis, and Care

**DOI:** 10.3389/fimmu.2017.00678

**Published:** 2017-06-26

**Authors:** Hamoud Al-Mousa, Bandar Al-Saud

**Affiliations:** ^1^Department of Pediatrics, King Faisal Specialist Hospital & Research Center, Riyadh, Saudi Arabia; ^2^Department of Genetics, King Faisal Specialist Hospital & Research Center, Riyadh, Saudi Arabia; ^3^College of Medicine, Alfaisal University, Riyadh, Saudi Arabia

**Keywords:** immunodeficiency, Middle East, North Africa, consanguinity, primary immunodeficiency, SCID, hematopoietic stem cell transplantation

## Abstract

Middle East and North Africa region (MENA)^1^ populations are of different ethnic origins. Consanguineous marriages are common practice with an overall incidence ranging between 20 and 50%. Primary immunodeficiency diseases (PIDs) are a group of heterogeneous genetic disorders caused by defects in the immune system that predisposes patients to recurrent infections, autoimmune diseases, and malignancies. PIDs are more common in areas with high rates of consanguineous marriage since most have an autosomal recessive mode of inheritance. Studies of PIDs in the region had contributed into the discovery and the understanding of several novel immunodeficiency disorders. Few MENA countries have established national registries that helped in estimating the prevalence and defining common PID phenotypes. Available reports from those registries suggest a predominance of combined immunodeficiency disorders in comparison to antibody deficiencies seen in other populations. Access to a comprehensive clinical immunology management services is limited in most MENA countries. Few countries had established advanced clinical immunology service, capable to provide extensive genetic testing and stem cell transplantation for various immunodeficiency disorders. Newborn screening for PIDs is an essential need in this population considering the high incidence of illness and can be implemented and incorporated into existing newborn screening programs in some MENA countries. Increased awareness, subspecialty training in clinical immunology, and establishing collaborating research centers are necessary to improve patient care. In this review, we highlight some of the available epidemiological data, challenges in establishing diagnosis, and available therapy for PID patients in the region.

## Introduction

Primary immunodeficiency diseases (PIDs) are a group of inherited heterogeneous disorders caused by monogenetic immune defects that predispose patients to infections (1). In addition, PID patients have non-infectious manifestation related to disturbed immune regulation that might cause lymphoproliferative and/or autoimmune manifestations (2). In 1952, Bruton described the first case of agammaglobulinemia (3). Since then, over 300 forms of PIDs have been described and characterized. The International Union of Immunological Societies PIDs Classification Committee (4) classified PID in to immunodeficiencies affecting cellular and humoral immunity, combined immunodeficiencies with associated or syndromic features, predominantly antibody deficiencies, disease of immune dysregulation, congenital defects of phagocyte number, function, or both, defects in intrinsic and innate immunity, autoinflammatory disorders, complement deficiencies, or phenocopies of PID. PIDs are considered to be rare disorders. Worldwide databases have shown geographical and racial variation in the epidemiology of PIDs. Published data from highly consanguineous population’s like the Middle East/Northern Africa (MENA) region showed that PIDs are not uncommon. A consanguineous marriage is usually defined in clinical genetic as a marriage between two couples who are second cousins or closer (5). Consanguineous marriages are common practice in MENA region with an overall incidence ranging between 20 and 50% (6) [Bittles A. H. and Black M. L. (2015) Global Patterns & Tables of Consanguinity http://consang.net]. This has provided a background where autosomal recessive (AR) diseases are abundant. For example, there are 955 genetic diseases that have been identified in Arabs from the MENA region, of which 586 (60%) are reported to be recessive diseases (7). In addition to high rates of consanguinity, the large family size and the rapid population growth all are factors responsible for the high prevalence of rare genetic diseases in the MENA region (8). Here, we present a review of PIDs status in a highly consanguineous population from the MENA region with particular emphasis on epidemiology, diagnosis, and care.

## MENA Definition, Population, and Ethnicity

The MENA region covers a surface area of nearly 15 million square kilometers from Morocco in the west to Iran in the east. The MENA region includes 22 countries and territories and accounts for 385 million people representing 6% of the world’s population (9). The MENA region has an annual population growth rate of 1.8% compared to a 1.2% average global population growth rate (United Nations, Department of Economic and Social Affairs-Population Division, Population Estimates and Projections Section-World Population Prospects, 2015 Revision). The pediatric age group (0–14 years) represents 31.1% of the total population in the MENA region in comparison to 26.1% globally. The MENA population has a mix of Asian, Caucasian, Arab, and African racial ancestries. MENA region captures pan-ethnic geographically defined groups that include Arab, Persian, Turkish, Kurdish, Berber, Amazigh, Assyrian, Chaldean, Armenian and others.

## Consanguinity

20% of world populations live in countries with a preference for consanguineous marriages (6). Among these, are the MENA region countries where consanguineous marriage is a normal practice for multiple sociocultural factors (10–15). The global consanguinity rate is 1–9% while it is 20–56% in the MENA region (4). PID population from the MENA region display a higher rate of consanguinity compared to their general population. In addition, AR inheritance is predominant. The T-B-NK+ SCID represents the most common SCID phenotype in 90, 87, and 50% of Saudi Arabia, Kuwait, and Egypt SCID patients, respectively. Family history suggestive of PID is common among patients in MENA region as captured in several registries at a rate of 30, 44, 48, 61, and 80% in Tunisia, Oman, Iran, Saudi Arabia, and Egypt, respectively (16–24). Moreover, a significant number of these AR PIDs were first described in patients living in the MENA region (25).

## Epidemiological Data and Registries

**Figure 1:**
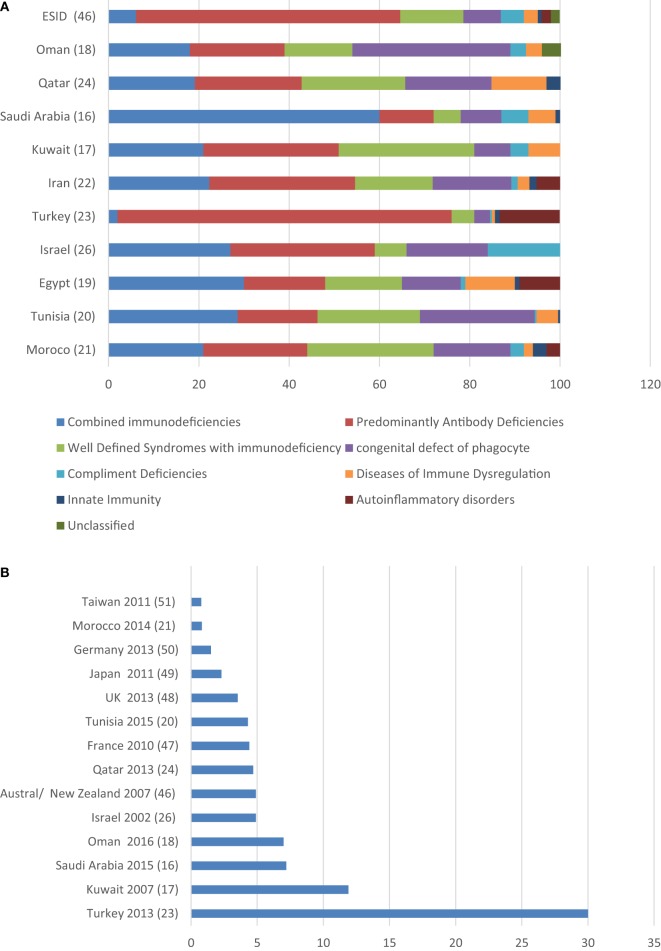
(A) Distribution of primary immunodeficiency disorders in different primary immunodeficiency disease registries from the MENA region compared to the European Society for Immunodeficiencies registry. (B) Primary immunodeficiency disease prevalence in 100,000 inhabitants in the MENA region compared with counties from other world region.

Ten countries from the MENA region have published epidemiological data in medical literature. These data result from a national registry or survey as those from Morocco (21), Tunisia (20), Israel (26), Kuwait (17), and Iran (22), or from a major referral centers in; Egypt (19), Turkey (23), Saudi Arabia (16), Qatar (24), and Oman (18). A total number of 4,918 patients were included. The male to female ratio ranged (1.1 to 2). The overall prevalence of PID was ranging from 0.81 to 30.5 in 100,000 populations (Table 1). In most registries more than 80% of patients were diagnosed at pediatric age group. The mean age at symptoms onset was within the first 2 years of life, except in Turkey, where it was at 4 years of age. That is probably explained by the fact that 70% of the patients of the Turkish registry are from a subgroup of predominantly antibody deficiencies while combined immunodeficiencies are commoner among other registries. Family history of PID was documented in seven registries and ranged from 17.2 to 61% (Table 1). Eight main PID categories based on IUIS classification were captured in various MENA registries as shown in Figure 1A. The disease distribution was variable among registries from the different MENA countries, where combined immunodeficiencies were the predominant category among patients registered in Saudi Arabia, Kuwait, Iran, Egypt, Israel, Tunisia, and Morocco. Predominantly antibodies deficiency was noticeably the commonest PID type among patients registered in Turkey. In addition, other PID categories were variably distributed in the registries, as, for example, congenital defect of phagocyte had a higher percentage of registered patients in both Oman and Tunisia while autoinflammatory disorder was higher in Turkey and Egypt in comparison to the other countries (Figure 1B).

**Table 1:** Patient’s characteristics from MENA region primary immunodeficiency disease registries.

Country (reference)	Source of cases (no. of sources)	Period (number of years)	Number of patients	Prevalence/100,000	Gender ratio male: female	Pediatrics age at diagnosis (%)	Mean age in months at onset of symptoms	Mean age in months at diagnosis	Family history (%)
Morocco (21)	National	1998–2012 (15)	421	0.81	1.17	94.1	16.8	74.5	19.1
Tunisia (20)	National	1988–2012 (25)	710	4.3	1.4	N/A	6 (median)	24 (median)	30
Egypt (19)	Center (1)	2010–2014 (5)	476	N/A	1.2	83	13	39	80
Israel (26)	National	1994–2000 (6)	294	4.9	2	82	N/A	N/A	N/A
Turkey (23)	Center (2)	2004–2010 (6)	1,435	30.5	1.6	94	49	62	N/A
Iran (22)	National	2006–2013 (7)	731	Incidence (9.7/1 million/year)	1.6	89	12 (median)	42 (median)	17.2
Kuwait (17)	National	2004–2006 (2)	76	11.9	1.6	98	15	43	43
Saudi Arabia (16)	Center (1)	2010–2013	504	7.2	1.1	93	17	39	61
Qatar (24)	Center (1)	1998–2012 (15)	131	4.7	1.3	N/A	24	42	N/A
Oman (18)	Center (1)	2005–2015 (10)	140	7	1.6	N/A	23	46	44

## Diagnostic Facilities

The extreme clinical, phenotypic, and genetic heterogeneity of many patients with PIDs represents significant diagnostic challenges to physicians in the MENA region. The capability to perform extensive immunologic investigations and mutation analysis is variable from one country to the other. In Saudi Arabia, an advanced clinical diagnostic immunology service at King Faisal Specialist Hospital and Research Centre (KFSHRC) had been established in the last 30 years that allowed access to diagnose most of the known PIDs. Extensive genetic testing that includes Sanger sequencing, Targeted next-generation sequencing PID gene panel, and whole exome sequencing is offered to affected patients (16, 27, 28). Sultan Qaboos University Hospital, the Royal Hospital, and Hamad General Hospital are the main PID centers in Oman and Qatar, respectively. AlSabah, Mubarak Al-Kabeer, and AlRahsid hospitals have PID centers in Kuwait. Genetic testing is performed through international diagnostic laboratories or through collaborative research facilities. The first immunology center in Turkey was established in 1972 at Hacettepe University in Ankara. There are now more than 10 immunology centers in different cities around Turkey with facilities to diagnose common PIDs (29). Departments of Pediatric Immunology at Uludag University Medical Faculty and Ege University Medical Faculty are the major centers reporting PID patients to European Society for Immunodeficiencies database from Turkey (23). Targeted next-generation sequencing PID genes panel and whole exome sequencing are offered to patients in Turkey. In Iran, there is at least one Medical University and immunology center at each of the 30 states of the country that could manage PIDs. National PID network is active in Iran, which allowed referring patients to the Research Center for Immunodeficiencies at Tehran University of Medical Sciences for further immunological and genetic testing (22). Queen Rania Children Hospital is the major pediatric immunology center in Jordan. In Lebanon, PID care is provided primarily by Pediatric infectious disease service at the American University of Beirut Medical center and few other medical centers. Several medical centers provide care and have diagnostic facilities to diagnose various types of PIDs in Israel with access to Sanger and whole exome sequencing (30). The Pasteur Institute of Tunisia is the main diagnostic facility in Tunisia with the ability to perform genetic testing for common PIDs. Ain Shams University center and Cairo University center are the main PID referral centers in Egypt; both are capable to diagnose major PID types while genetic testing is done at research diagnostic facilities in USA. In Algeria, there are five medical centers capable to diagnose and manage major types of PIDs. All are located in northern part of the country. Genetic testing for agammaglobulinemia, LAD, and MHC II deficiency is available and collaborates with research centers in France and USA to diagnose atypical PIDs. In other MENA countries, access is often limited because of either financial or personnel limitations.

## PID Care and Management

Specialist clinical immunology services and hematopoietic stem cell transplantation (HSCT) facilities emerged in a few countries driven by the health demands of the population. In Saudi Arabia, an HSCT program for PID had been established in 1984 at KFSHRC in Riyadh. More than 500 PID cases had been transplanted with an average of 35–40 transplants per year (16, 31–33). 70% of HSCTs are from HLA matched related donors whereas the remaining donor sources were either unrelated umbilical cord blood or haploidentical bone marrow. Another two centers recently started performing HSCT for PID at National Guard Hospital, Riyadh and KFSHRC, Jeddah. There are five centers performing HSCT for PID in Turkey, in Ankara (Hacettepe and Ankara University.), in Izmir (Ege University), in Antalya and in Istanbul (Medical Park Hospital). In Iran, there are two HSCT centers in Tahran (Hematology, Oncology and Stem Cell Transplantation Research Centre and Children’s Medical Center), and more than 50 HSCTs for PIDs had been performed over last 10 years. In Jordan, Queen Rania Children Hospital is the main transplanting center with an average of 8–10/year in addition to King Hussein Cancer Center (34). HSCTs are offered at several medical centers in Israel. Twenty eight HSCTs had been performed for PIDs in the last 8 years at bone marrow transplantation center of Tunis (27 genoidentical and 1 haploidentical). Sultan Qaboos University hospital in Oman performs two to four HSCTs for PID per year. A few HSCTs trials had been performed in Algeria, Egypt, and Morocco. Access to immunoglobulin replacement therapy and HSCT is very limited or unaffordable for many patients in some of the MENA countries.

## Novel PID Discovery

In the last three decades, more than 300 novel PIDs were discovered, and the list is continuously expanding. The inbred population of the MENA region provides a great opportunity to identify monogenic PIDs through novel next-generation sequencing technology. Studies of PIDs in the region had contributed into the discovery and the understanding of large numbers of these disorders. For example, more than 12 novel PID genes were discovered through studying patients from the MENA region in the last 2 years, which include DOCK2 (35), HOIP (36), IL-17RC (37), RORC (38), RLTPR (39), POLE2 (40), NEIL3 (41), TFRC (42), INO80 (43), LAT (44), MYSM1 (45), and CD70 (46).

## Genetic Counseling and Disease Prevention

In the MENA countries, the majority of patients has an AR mode of inheritance and come from families known to have the disease. Appropriate genetic counseling for affected families is an essential part of the management. In Saudi Arabia, Turkey, Iran, Israel, and Kuwait genetic counseling, prenatal and preimplantation genetic diagnosis and premarriage screening to identify carriers are offered to affected families. Such services are costly and require sophisticated diagnostic facilities not available in most of the MENA countries.

## Education and Training

Establishing a structural immunology educational and fellowship programs is essential to improve available immunology services in MENA region. In Saudi Arabia, allergy and immunology fellowship training program had been established since 1989. Structured clinical immunology training had been also established in Iran 30 years ago. A 3-year fellowship training is available in several universities in Turkey. Allergy and immunology fellowship program had been recently launched in Kuwait. Immunological centers at MENA region are actively involved in providing education sessions and training for general practitioners, pediatricians, and internists. Dedicated conferences and workshops on PID had been organized in various MENA countries aiming to increase the health providers’ awareness.

## Newborn Screening for PID

Most patients with PIDs are asymptomatic at birth. Early diagnosis and initiation of therapy improve the outcome. SCID have been recognized as candidates for population-based newborn screening using the T-cell receptor recombination excision circle assay (TREC) and found to be cost-effective means to improve the quality and duration of life for children with SCID. The high disease incidence as seen in MENA region is a critical driving force that would affect the incremental cost-effectiveness ratio. Implementing such a program in the MENA region is possible in resource-rich countries. However, the health authorities should recognize the seriousness of the health problem and provide all required resources. Israel is the only MENA country performing universal SCID NBS. A NBS pilot study is ongoing in Saudi Arabia to identify the real incidence of SCID in Saudi population.

## Discussion

The MENA region has a diverse ethnic background, but consanguineous marriages are common practice among this population. This practice is derived by cultural and socioeconomic interests aiming to strengthen family relations and structure. PIDs are more common in areas with high rates of consanguineous since most PIDs are inherited in an AR pattern and hence the observed increased incidence of combined immunodeficiencies among MENA populations in comparison to populations from the European countries (47) (Figure 1A). The higher incidence of combined immunodeficiency seen in Saudi Arabia in comparison to other MENA countries might be related to the availability of diagnostic facilities and to the fact that this was a single center registry. The lower rate of consanguineous marriage in Turkey had contributed to the reduced incidence of such disorders in their population. Moreover registries from the MENA region (16–21, 23, 24, 26) showed a high prevalence of PIDs when compared to the rest of the world (48–53) (Figure 1B). The variable low or high PID reported prevalence might be a reflection of the accessibility and the availability for diagnostic facilities in this population among MENA countries.

The majority of patients with PID in MENA region have a family history suggestive of PID. It would be cost effective to establish a family-based preventive program for PID in this region, which allows identifying all family carriers, offer genetic counseling, and premarriage screening. Affected couples can be offered preimplantation or prenatal genetic diagnosis. However, such program should respect social and religious beliefs.

A lot of excitements are observed among clinicians taking care of PID in MENA region, but they are confronted with lack of specialized centers dedicated to the care of PID patients, limited access to a diagnostic facilities and the costly burden for the therapeutic modalities. Molecular genetic testing is essential diagnostic tool for PIDs, it provides a definitive diagnosis, assists in genetic counseling, establishes the diagnosis in atypical cases, provides genotype–phenotype correlation and guide therapy for affected patients. Molecular genetic testing is complex, expensive, and unaffordable in most of MENA countries. It will be important to establish multicenter collaborations in the region to provide and share diagnostic facilities and therapy. This will require strategic support from heath authorities in the region. Governmental support and charity funding are essential to establish clinical services and ensure that patients can receive long term therapy such as immunoglobulins replacement therapy or stem cell transplantation.

Establishment of a MENA primary immunodeficiency network is essential to promote collaborations and experience sharing. Collaboration with various international research centers through implementing advanced molecular genetic testing had led into several novel genetic discoveries in the field of PID. Multicenter collaborations especially between clinicians and basic scientists can contribute in development of efficient PID research in the region. Clinicians’ training is important to promote PID care in MENA region. This can be enforced through including clinical immunology in the undergraduate and postgraduate educational curriculum, implementing formal subspecialty training for pediatric and adult immunologist and through providing continuous medical education courses in clinical immunology.

This report highlights the need to improve awareness about PID in MENA region, enhances structural training in clinical immunology, and establishes national registries and centers of excellence in PID care and stem cell transplantation.

## Author Contributions

HA-M and BA-S participated in data collection, analysis and interpretation, and drafting and final approval of manuscript.

## Conflict of Interest Statement

The authors declare that the research was conducted in the absence of any commercial or financial relationship that could be construed as a potential conflict of interest.
